# Conventional versus modified nipple sparing mastectomy in immediate breast reconstruction: Complications, aesthetic, and patient-reported outcomes

**DOI:** 10.3389/fsurg.2022.1001019

**Published:** 2022-09-21

**Authors:** Bakhtiyor Najmiddinov, Joseph Kyu-hyung Park, Kyung-Hwak Yoon, Yujin Myung, Hyoung Won Koh, Ok Hee Lee, Jeong Jae Hoon, Hee Chul Shin, Eun-Kyu Kim, Chan Yeong Heo

**Affiliations:** ^1^ Department of Plastic and Reconstructive Surgery, Seoul National University Bundang Hospital, Seongnam, South Korea; ^2^ Department of Surgery, Seoul National University Bundang Hospital, Seongnam, South Korea

**Keywords:** breast cancer, breast reconstruction, mastectomy, patient reported outcome, postoperative complications

## Abstract

**Background:**

Nipple-sparing mastectomy (NSM) followed by immediate breast reconstruction (IBR) is the optimal surgical treatment for breast cancer. However, investigations are ongoing to improve the surgical technique to achieve better results. This study aimed to evaluate the outcomes of modified NSM (m-NSM), which preserves the anterior lamellar fat layer, in patients who underwent IBR.

**Methods:**

All patients who underwent modified NSM (m-NSM) or conventional NSM (c-NSM) followed by IBR using autologous tissue or implants were retrospectively reviewed between January 2014 and January 2021. Two mastectomy types were compared in terms of postoperative complications and aesthetic outcomes using panel assessment scores by physicians and reported outcomes using Breast-Q. In addition, postoperative evaluations of the thickness of mastectomy flap was performed using CT scan images.

**Results:**

A total of 516 patients (580 breasts) with NSM (143 breasts with c-NSM and 437 breasts with m-NSM) followed by IBR were reviewed. The mean ± SD flap thickness was 8.48 ± 1.81 mm in patients who underwent m-NSM, while it was 6.32 ± 1.15 mm in the c-NSM cohort (*p* = 0.02). The overall major complications rate was lower in the m-NSM group (3.0% vs. 9.0%, *p *< 0.013). Ischemic complications of the mastectomy flap and nipple-areolar complex (NAC) were more in c-NSM, although the difference was not statistically significant. The mean panel assessment scores were higher in the m-NSM group (3.14 (good) and 2.38 (fair) in the m-NSM and c-NSM groups, respectively; *p *< 0.001). Moreover, m-NSM was associated with greater improvements in psychosocial (*p *< 0.001) and sexual (*p *= 0.007) well-being.

**Conclusion:**

Preserving the anterior lamellar fat in NSM was associated with thicker mastectomy flap, overall lower rates of complications, including ischemia of the mastectomy flap and nipple-areolar complex, and was associated with better aesthetic outcomes and improved quality of life.

## Introduction

There has been considerable improvement in breast cancer therapy and overall survival rates due to advances in early stage detection and targeted therapies over the last decade. Highly improved outcomes were obtained as mastectomy techniques shifted from more radical treatments with the regular removal of the nipple-areolar complex (NAC) to less extensive and personalized modalities. The patients-driven demand for continuous improvements in cosmesis has led surgeons to consider performing nipple-sparing mastectomy (NSM), which preserves almost the entire breast skin envelope and NAC while removing the glandular and ductal tissues (1, 2).

As the NAC defines the breast and provides its identity (3), the NSM produces enhanced cosmetic results during breast reconstruction by retaining the NAC and most of the breast skin envelope. According to Didier et al., patients showed a high level of satisfaction with nipple preservation and consider NSM as beneficial in helping them cope with the painful experiences of breast cancer and breast loss (4). In 78.6% of cases, patient satisfaction with NSM was good to excellent, and 42.9% of patients retained nipple sensation (5). Currently, it is the gold standard for treating patients with oncologically suitable breast cancer (6–8). Nipple-sparing mastectomy (NSM) followed by immediate breast reconstruction (IBR) using implants or autologous tissue is considered the most optimal surgical treatment option for selected patients with breast cancer, facilitating superior aesthetic outcomes and improved quality of life (QOL) (9, 10). Aesthetic results following NSM and IBR have been reported to be good to excellent in 75–90 percent of patients (1).

However, ischemic complications ranging from partial to full NAC and/or mastectomy flap necrosis are frequent adverse events that affect the overall outcomes of reconstruction and patient satisfaction after NSM (11–13). Preservation of the superficial vasculature in the subdermal and subcutaneous tissues, which perfuses the NAC and the skin flap, is crucial to reducing ischemic complications. The modified NSM (m-NSM) was used to reduce postoperative complications while improving the aesthetic and patient-reported outcomes by preserving the anterior lamellar fat layer, which increases the thickness and perfusion of the mastectomy flap.

This study aimed to analyze m-NSM outcomes in patients who underwent IBR. Complications rate, aesthetic outcomes using panel assessment scores by physicians, and patient-reported outcomes using Breast-Q in a large number of patients were examined. In addition, postoperative thickness of mastectomy flap was measured using CT scan images. To our knowledge, this is the first study that measured the mastectomy flap thickness using CT scan.

## Materials and methods

### Data collection

Written informed consent was obtained from all the patients before surgery. The study was approved by the Institutional Review Board of Seoul National University Bundang Hospital (IRB No. B-2112-724-102). Electronic medical records of patients who underwent NSM followed by IBR were reviewed retrospectively at the Seoul National University Bundang Hospital (SNUBH) between January 2014 and January 2021. Detailed patient demographics, oncologic and reconstruction-related data, and medical records were reviewed using our institutional hospital database. Baseline patient characteristics, such as age, body mass index, hypertension, active smoking, and diabetes, were examined. Patients with NAC or skin involvement, inflammatory cancer, Paget's disease, stage IV breast cancer at initial presentation, and any other mastectomy types and delayed reconstruction were excluded from this study. We divided all included patients into two groups according to the mastectomy method: (1) conventional NSM (c-NSM) followed by IBR, and (2) modified NSM (m-NSM) followed by IBR. The two surgical methods are classified according to the surgeon who performed the operation. Two surgeons have performed the c-NSM method, and one has operated using the m-NSM method. Therefore, we were able to perform the analysis by clearly classifying the surgical method in the retrospective analysis. IBR was done by two plastic surgeons. Due to the low number of patients with post-mastectomy radiation therapy (PMRT), we included only patients who did not receive PMRT to avoid bias.

### Surgical techniques

#### Conventional NSM (c-NSM)

After the skin incision was performed, skin flaps were elevated to the sternum medially to the latissimus dorsi laterally, clavicle superiorly, and costal margin inferiorly with the Bovie coagulator on the superficial fascial plane anteriorly. Skin flaps were developed along the superficial layer of superficial fascia, which results in an even flap thickness throughout the whole breast. The plane between the pectoralis major fascia and pectoralis major muscle was the posterior plane of dissection.

#### Modified NSM (m-NSM)

**Figure 1:**
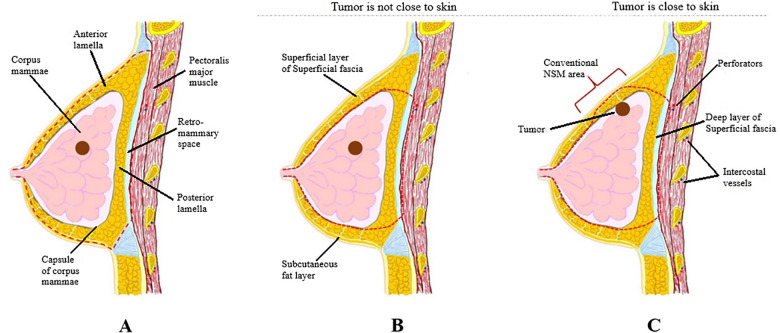
(**A**) Dissection plane in c-NSM: a dissection plane is on the superficial fascial plane, and the plane between pectoralis major fascia and muscle is a posterior plane of dissection; (**B**) dissection plane in m-NSM when the tumor is not close to breast capsule: anterior dissection plane is on breast capsule and posterior dissection plane is between pectoralis major fascia and muscle; (**C**) dissection plane in m-NSM when the tumor is in contact with the capsule of corpus mammae: anterior dissection plane is on superficial margin near the tumor which is same with c-NSM. c-NSM, conventional nipple-sparing mastectomy; m-NSM, modified nipple-sparing mastectomy.

**Figure 2:**
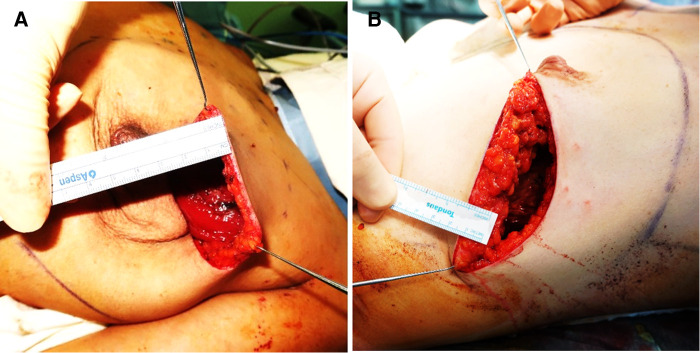
Images show the intraoperative thickness of mastectomy flaps: (**A**) 42 years old patient with left breast cancer presented with c-NSM (BMI:23.61 flap thickness = 0.5 cm); (**B**) 39 years old patient diagnosed with right breast cancer is shown after m-NSM (BMI:25.21; flap thickness = 1.5 cm). c-NSM, conventional nipple-sparing mastectomy; m-NSM, modified nipple-sparing mastectomy.

A skin incision followed by dissection to the sternum medially, clavicle superiorly, latissimus dorsi muscle laterally, and costal margin inferiorly. The main difference between m-NSM and c-NSM is the anterior plane of dissection, which is performed along with the breast capsule, the anterior capsule of corpus mammae. The breast parenchyma is separated from the subcutaneous fat layer by the breast capsule, representing the anatomic dissection plane. This dissection plane can maximize the preservation of the anterior lamellar fat layer, which increases the thickness of the mastectomy flap. When the tumor is close to the breast capsule, dissection above the tumor area is performed along the superficial layer of superficial fascia as in c-NSM to ensure oncological safety. The posterior dissection plane was the same as the c-NSM, which was dissected under the pectoralis major muscle fascia. Graphical illustrations of the dissection planes in both the c-NSM and m-NSM are shown in Figure 1. Regardless of the mastectomy type, sharp dissection was performed in all patients, minimizing the application of electrocautery limited to hemostasis to prevent thermal damage to the mastectomy flap. Intraoperative images of the mastectomy flap immediately after c-NSM and m-NSM are shown in Figure 2.

The patients undergoing either mastectomy types were evaluated intraoperatively right after the mastectomy using ICG (Indocyanine green) angiography (Fluobeam®, Fluoptics) for the quality of perfusion before the IBR is performed.

#### Immediate breast reconstruction (IBR)

**Figure 3:**
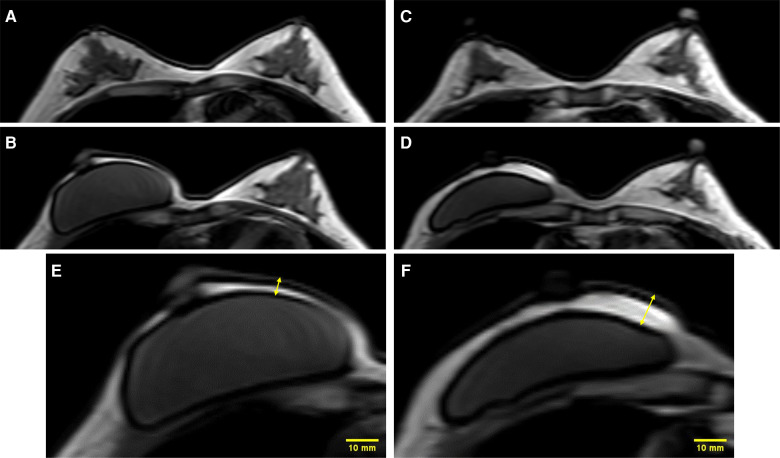
Difference in mastectomy flap thickness between c-NSM and m-NSM on MRI images: (**A**) preoperative MRI image of the patient with right breast cancer; (**B**) one-month postoperative MRI image of the same patient after c-NSM and IBR using implant; (**C**) preoperative MRI image of another patient with right breast cancer; (**D**) postoperative MRI image after m-NSM and IBR using implant at 1-month follow-up; (**E**) 2x image of the breast shows the flap thickness of 4.92 mm after c-NSM; (**F**) flap thickness was 10.23 mm after m-NSM. c-NSM, conventional Nipple Sparing Mastectomy; m-NSM, modified Nipple Sparing Mastectomy.

Immediate autologous or implant-based reconstruction was performed based on the preoperative plan, depending on the desires of the patients and the availability of donor sites. A free muscle-sparing transverse rectus abdominis myocutaneous flap (MS-TRAM) or a pedicled latissimus dorsi (LD) flap was transferred as an autologous reconstruction modality, while implant-based reconstruction was performed either in a single stage using a silicone implant or staged reconstruction using a tissue expander followed by silicone implant insertion. The difference between c-NSM and m-NSM in flap thickness is shown in Figure 3 using preoperative and postoperative MRI images of the breast.

### Flap thickness of mastectomy flap

**Figure 4:**
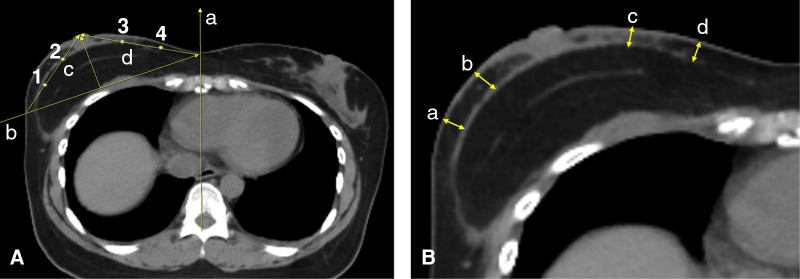
The graphical illustration of the flap thickness using CT scan: (**A**) (a) midsagittal line; (b) a line drawn from line “a” to the lateral pole of the breast through the outer surface of the rib cage; (c) a line connecting the center of the most projected point of the nipple to the crossing point of the line “b” with the breast skin at the lateral pole; (d) a line connecting the center of the most projected point of the nipple to the crossing point of the line “b” with the line “a” in the midline; (1) and (2) points that divide the line “c” into three equal lines; (3) and (4) points that divide the line “d” into three equal lines; (**B**) (a) and (b) the flap thickness measurement points on the lateral pole of the breast; (c) and (d) the point of flap thickness measurement on the medial pole of the breast.

We have measured the flap thickness after c-NSM and m-NSM followed by IBR using postoperative axial CT scan images. Considering the postoperative swelling, we have measured the flap thickness in patients who have been followed up at least 1 year with available postoperative CT scan to avoid the inaccuracy. For this, the CT slice where the nipple is the most projected found. A midsagittal line (line “a”, Figure 4A) is drawn from the vertebral spine to the center of the sternum. Then, line “b” is drawn from point “a” in the midline to the lateral pole of the breast through the outer surface of the rib cage. “c” and “d” lines are marked from the center of the nipple to the line “b” on lateral and medial sides, respectively. Points 1 and 2 are located to divide the line “c” into equal thirds, while points 3 and 4 are marked along the line “d”. A mastectomy thickness is measured at points 1,2,3 and 4. Figure 4B shows the mastectomy thickness measurement points. The average value of the four measured points was calculated.

### Complications

We analyzed the postoperative complications (NAC or mastectomy flap necrosis, wound healing problems, seroma, reconstruction failure, implant rippling, animation deformity, hematoma, infection, etc.) that occurred in the early phase until 6 months after surgery and compared the complication rates between the groups. Reconstructive failure was defined as flap loss in the autologous reconstruction group, an unplanned, non-aesthetic tissue expander/implant removal due to a complication in the expander/implant cohort. Issues that did not necessitate surgical intervention were defined as minor complications, while those managed surgically were considered major.

### Aesthetic outcomes

A five-member panel assessment scoring system comprising two plastic surgeons and three general surgeons rated the aesthetic results. Operating surgeons were not involved in panel assessment scoring. The raters were provided with blinded data consisting of patients’ frontal, oblique, and lateral view digital images and were asked to rate using the Harvard scale (14). The panel was not provided any details regarding the mastectomy and reconstruction methods. Only results from above the umbilicus to the shoulders were examined. Only images taken 6–12 months postoperatively were included for the assessment. The scores ranged from 1 to 4, with 4 points representing excellent, 3-good, 2-fair, and 1-poor aesthetic results, respectively.

### Quality of life (QOL)

A survey was conducted using the reconstruction module of Breast-Q Version 2.0 (Copyright ©2012, Memorial Sloan Kettering Cancer Center, and The University of British Columbia) postoperative scales. The questionnaire was sent to all patients who underwent either c-NSM or m-NSM followed by IBR using either implant or autologous tissue, and patients with incomplete or missing answers were excluded from the analysis. The reconstruction module of the Breast Q version 2.0 consists of questions grouped into health-related quality of life (QOL) and patient satisfaction. Although answers were received to all questions, only acceptable questions were used for the analysis. The psychosocial, sexual, and physical well-being scores were analyzed from the QOL domain, while the scores for satisfaction with breast, information, and the surgeon were included from the patient satisfaction domain. License was obtained, and using the corresponding scoring table, values for BREAST-Q version 2.0 were converted to the equivalent Rasch transformed scores, which ranged from 0 to 100, with higher scores indicating better QOL or greater satisfaction.

### Statistical analysis

Categorical variables were analyzed using Fisher's exact test with Monte Carlo simulation with 2,000 replicates, and the Kruskal-Wallis test was applied to analyze continuous variables. Kendall's W coefficient of concordance was used to estimate the inter-rater reliability in panel assessment scoring. All statistical analyses were performed using R (version 4.0.1; R Foundation for Statistical Computing, Vienna, Austria) and RStudio (version 1.3.959; PBC, MA) (15), and *p* < 0.05 was considered statistically significant.

## Results

### Baseline characteristics

Among the 516 patients (580 breasts) that underwent NSM, c-NSM was performed in 143 breasts (131 patients), whereas 437 breasts (385 patients) underwent m-NSM. After excluding breasts with PMRT, 133 breasts with c-NSM and 330 with m-NSM were included. The mean age of the patients was similar in both groups (45.98 ± 6.77 vs. 45.59 ± 7.00 years old, *p *= 0.579). The mean value of BMI was 22.69 ± 2.89 in c-NSM, while it was 22.52 ± 3.02 in m-NSM (*p *= 0.301). The mean follow-up period in the c-NSM and m-NSM groups were 41.92 ± 21.62 and 31.98 ± 19.98 months, respectively (*p *< 0.001). The rates of diabetes mellitus (DM), hypertension, and smoking were not significantly different between the groups, with *p* values of 0.694, 0.08, and 0.124, respectively. All the included patients had unilateral breast cancer. The baseline characteristics of the patients are shown in Table 1.

**Table 1:** Patient characteristics by mastectomy type (*N* = 463).

Variable		Overall	c-NSM	m-NSM	*p*-value
*n*		463	133	330	
BMI, kg/m^2^ [mean (SD)]		22.56 (2.98)	22.69 (2.89)	22.52 (3.02)	0.301
Age, years [mean (SD)]		45.70 (6.93)	45.98 (6.77)	45.59 (7.00)	0.579
DM, *n* (%)	No	445 (96.1)	126 (94.7)	319 (96.7)	0.694
	Yes	8 (1.7)	3 (2.3)	5 (1.5)	
	NA	10 (2.2)	4 (3.0)	6 (1.8)	
HTN, *n* (%)	No	432 (93.3)	119 (89.5)	313 (94.8)	0.080
	Yes	21 (4.5)	10 (7.5)	11 (3.3)	
	NA	10 (2.2)	4 (3.0)	6 (1.8)	
Active smoking, *n* (%)	No	426 (92.0)	118 (88.7)	308 (93.3)	0.124
	Yes	35 (7.6)	14 (10.5)	21 (6.4)	
	NA	2 (0.4)	1 (0.8)	1 (0.3)	
Laterality, *n* (%)	Left	227 (49.0)	67 (50.4)	160 (48.5)	0.758
	Right	236 (51.0)	66 (49.6)	170 (51.5)	
Diagnosis, *n* (%)	DCIS	130 (28.1)	41 (30.8)	89 (27.0)	0.431
	IDC	252 (54.4)	73 (54.9)	179 (54.2)	
	ILC	15 (3.2)	5 (3.8)	10 (3.0)	
	LCIS	4 (0.9)	1 (0.8)	3 (0.9)	
	mixed	4 (0.9)	2 (1.5)	2 (0.6)	
	others	39 (8.4)	9 (6.8)	30 (9.1)	
	NA	19 (4.1)	2 (1.5)	17 (5.2)	
pT, *n* (%)	T0	38 (8.2)	4 (3.0)	34 (10.3)	0.052
	T1	219 (47.3)	67 (50.4)	152 (46.1)	
	T2	75 (16.2)	21 (15.8)	54 (16.4)	
	T3	4 (0.9)	0 (0.0)	4 (1.2)	
	Tis	127 (27.4)	41 (30.8)	86 (26.1)	
pN, *n* (%)	N0	359 (77.5)	109 (82.0)	250 (75.8)	0.390
	N1	38 (8.2)	10 (7.5)	28 (8.5)	
	N1mi	14 (3.0)	5 (3.8)	9 (2.7)	
	N2	11 (2.4)	3 (2.3)	8 (2.4)	
	N3	6 (1.3)	1 (0.8)	5 (1.5)	
	Nx	35 (7.6)	5 (3.8)	30 (9.1)	
AJCC stage, *n* (%)	0	140 (30.2)	41 (30.8)	99 (30.0)	0.3713
	I	5 (1.1)	3 (2.3)	2 (0.6)	
	IA	175 (37.8)	52 (39.1)	123 (37.3)	
	IB	13 (2.8)	6 (4.5)	7 (2.1)	
	IIA	70 (15.1)	19 (14.3)	51 (15.5)	
	IIB	23 (5.0)	5 (3.8)	18 (5.5)	
	IIIA	12 (2.6)	4 (3.0)	8 (2.4)	
	IIIC	6 (1.3)	1 (0.8)	5 (1.5)	
	NA	19 (4.1)	2 (1.5)	17 (5.2)	
ER, *n* (%)	Negative	72 (15.6)	18 (13.5)	54 (16.4)	0.399
	Positive	372 (80.3)	113 (85.0)	259 (78.5)	
	NA	19 (4.1)	2 (1.5)	17 (5.2)	
PR, *n* (%)	Negative	109 (23.5)	27 (20.3)	82 (24.8)	0.229
	Positive	335 (72.4)	104 (78.2)	231 (70.0)	
	NA	19 (4.1)	2 (1.5)	17 (5.2)	
Her2, *n* (%)	Negative	255 (55.1)	75 (56.4)	180 (54.5)	0.469
	Positive	84 (18.1)	21 (15.8)	63 (19.1)	
	Borderline	105 (22.7)	35 (26.3)	70 (21.2)	
	NA	19 (4.1)	2 (1.5)	17 (5.2)	
Ki-67, *n* (%)	<10%	203 (43.8)	61 (45.9)	142 (43.0)	0.835
	≥10%	241 (52.1)	70 (52.6)	171 (51.8)	
	NA	19 (4.1)	2 (1.5)	17 (5.2)	
Neoadjuvant CTx, *n* (%)	Not received	394 (85.1)	128 (96.2)	266 (80.6)	<0.001
	Received	69 (14.9)	5 (3.8)	64 (19.4)	
Adjuvant CTx, *n* (%)	Not received	438 (94.6)	122 (91.7)	316 (95.8)	0.109
	Received	25 (5.4)	11 (8.3)	14 (4.2)	
Neoadjuvant RTx, *n* (%)	Not received	419 (90.5)	124 (93.2)	295 (89.4)	0.225
	Received	44 (9.5)	9 (6.8)	35 (10.6)	
Adjuvant Hx, *n* (%)	Not received	88 (19.0)	22 (16.5)	68 (20.6)	0.412
	Received	375 (81.0)	111 (83.5)	262 (79.4)	
Follow-up, month [mean (SD)]		34.83 (20.93)	41.92 (21.62)	31.98 (19.98)	<0.001

c-NSM, conventional nipple sparing mastectomy; m-NSM, modified nipple sparing mastectomy; BMI, body mass index; SD, standard deviation; DM, diabetes mellitus; HTN, hypertension; pT, pathologic tumor stage; pN, pathologic node stage; AJCC, American joint committee on cancer; ER, esterogen receptor; PR, progesterone receptor; HER2, human epidermal growth factor receptor 2; CTx, chemotherapy; RTx, radiotherapy; Hx, hormone teraphy; NA, not available; DCIS, ductal carcinoma *in situ*; IDC, invasive ductal carcinoma; ILC, invasive lobular carcinoma; LCIS, lobular carcinoma *in situ*.

### Operative details

The commonly used mastectomy incisions were lateral radial, IMF, and inverted T incisions. All other incision patterns performed following previous breast-conserving surgery were classified as others. Importantly, periareolar incision was not used in our patients.

Of the 133 breasts that underwent c-NSM, 48 (36.1%) were reconstructed using tissue expanders, 26 (19.5%) underwent direct to implant (DTI) reconstruction, while autologous reconstruction was performed in 56 (42.1%) using free TRAM flap and in 3 (2.3%) breasts with pedicled LD flap. Tissue expanders were employed in 82 (24.8%) of the 330 breasts in the m-NSM group, DTI reconstruction in 82 (24.8%), free TRAM flap in 125 (37.9%), and pedicled LD flap in 41 (12.4%). Moreover, balancing procedures rates were significantly different (*p* < 0.001) between groups: 0.8% and 7.6% of patients underwent augmentation of the opposite breast, while mastopexy was performed in 2.8% and 5.3% in c-NSM and m-NSM groups, respectively, and the percentages of reduction mammoplasty were similar (1.5%) in both groups (Table 2). The mean weight of the specimen was 408.54 ± 161.39 in c-NSM group, while it was 279.23 ± 136.02 in m-NSM group (*p* < 0.05).

**Table 2:** Operative details by mastectomy type (*N* = 463).

	Level	Overall	c-NSM	m-NSM	*p*-value
*n*		463	133	330	
Incision, *n* (%)	Lateral radial	311 (67.2)	85 (63.9)	226 (68.5)	<0.001
	IMF	113 (24.4)	21 (15.8)	92 (27.9)	
	Inverted-T	2 (0.4)	1 (0.8)	1 (0.3)	
	Other	37 (8.0)	26 (19.5)	11 (3.3)	
Reconstruction, *n* (%)	TRAM	181 (39.1)	56 (42.1)	125 (37.9)	<0.001
	DTI	108 (23.3)	26 (19.5)	82 (24.8)	
	TEI	130 (28.1)	48 (36.1)	82 (24.8)	
	LD	44 (9.5)	3 (2.3)	41 (12.4)	
Opposite breast surgery, *n* (%)	Augmentation	26 (5.6)	1 (0.8)	25 (7.6)	<0.001*
	Reduction	7 (1.5)	2 (1.5)	5 (1.5)	
	Mastopexy	13 (2.8)	7 (5.3)	6 (1.8)	

c-NSM, conventional nipple sparing mastectomy; m-NSM, modified nipple sparing mastectomy; IMF, inframammary fold; TRAM, transverse rectus abdominis myocutaneous; DIEP, deep inferior epigastric perforator; SGAP, superior gluteal artery perforator; DTI, direct-to-implant; TEI, tissue expander insertion; LD, latissimus dorsi.

### Oncologic safety

The patients included in the c-NSM and m-NSM groups were similar in diagnosis, tumor and axillary lymph node status, AJCC stage, ER status, PR status, HER2 status, and Ki-67 status (Table 1). During the average follow-up period of 34.83 months (41.92 and 31.98 months for c-NSM and m-NSM groups, respectively), 10 recurrence events were observed (3% in c-NSM and 1.8% in m-NSM group, *p *= 0.49). The local recurrence rates were 1.5% and 0.3% in c-NSM and m-NSM, respectively (*p *= 0.209), while one patient (0.8%) had a distal recurrence in the liver in c-NSM and one patient (0.3%) had a recurrence in the endometrium in the m-NSM group (*p *= 0.504) (Table 3).

**Table 3:** Recurrence rate.

		Overall	c-NSM	m-NSM	*p-*value
*n*		463	133	330	
Overall recurrence, *n* (%)	No	434 (93.7)	127 (95.5)	307 (93.0)	0.490*
	Yes	10 (2.2)	4 (3.0)	6 (1.8)	
	NA	19 (4.1)	2 (1.5)	17 (5.2)	
Local recurrence, *n* (%)	No	441 (95.2)	129 (97.0)	312 (94.5)	0.209*
	Yes	3 (0.6)	2 (1.5)	1 (0.3)	
	NA	19 (4.1)	2 (1.5)	17 (5.2)	
Distant recurrence, *n* (%)	No	442 (95.5)	130 (97.7)	312 (94.5)	0.504*
	Yes	2 (0.4)	1 (0.8)	1 (0.3)	
	NA	19 (4.1)	2 (1.5)	17 (5.2)	

c-NSM, conventional nipple sparing mastectomy; m-NSM, modified nipple sparing mastectomy.

### Complications

The rate of major complications in all included breasts that required surgical intervention was 9% in c-NSM and 3% in m-NSM (*p *= 0.013), whereas minor complications were 17.3% and 11.2%, respectively (*p *= 0.092). A partial mastectomy flap necrosis occurred in 1.5% of c-NSM breasts, but no flap necrosis was observed in the m-NSM group (*p *= 0.082). The c-NSM group presented with 2.3% partial and 0.8% total NAC necrosis, while partial necrosis occurred in 0.3% (*p* = 0.074) breasts without the total NAC necrosis in the m-NSM group (*p *= 0.287). The rates of wound-healing-related complications (*p *= 0.023) and implant rippling (*p *= 0.06) were higher in the c-NSM group. Detailed data on complications by mastectomy type are presented in Table 4. To see the differences in terms of reconstruction modalities, we have compared the complications by reconstruction methods. No statistically significant difference in terms of reconstruction methods was found in c-NSM cohort (Supplementary Table S4-1), while in m-NSM group, the rate of seroma was higher in TRAM and TEI reconstructions (*p *= 0.024), the rate of reconstruction failure was higher in DTI and TEI reconstructions (*p *= 0.049), and the rate of infection was higher in TRAM and TEI reconstructions (*p *= 0.042) (Supplementary Table S4-2). The analysis in terms of autologous versus implant-based reconstruction did not show any statistically significant differences (Supplementary Tables S4-3, S4-4).

**Table 4:** Complication by mastectomy type (*N* = 463).

	Level	Overall	c-NSM	m-NSM	*p*-value
*n*		463	133	330	
Partial skin necrosis, *n* (%)	No	461 (99.6)	131 (98.5)	330 (100.0)	0.082
	Yes	2 (0.4)	2 (1.5)	0 (0.0)	
Partial NAC necrosis, *n* (%)	No	459 (99.1)	130 (97.7)	329 (99.7)	0.074
	Yes	4 (0.9)	3 (2.3)	1 (0.3)	
Total NAC necrosis, *n* (%)	No	462 (99.8)	132 (99.2)	330 (100.0)	0.287
	Yes	1 (0.2)	1 (0.8)	0 (0.0)	
Wound healing problem, *n* (%)	No	456 (98.5)	128 (96.2)	328 (99.4)	0.023
	Yes	7 (1.5)	5 (3.8)	2 (0.6)	
Seroma, *n* (%)	No	458 (98.9)	131 (98.5)	327 (99.1)	0.628
	Yes	5 (1.1)	2 (1.5)	3 (0.9)	
Reconstruction failure, *n* (%)	No	449 (97.0)	124 (93.2)	325 (98.5)	0.005
	Yes	14 (3.0)	9 (6.8)	5 (1.5)	
Implant rippling, *n* (%)	No	457 (98.7)	129 (97.0)	328 (99.4)	0.06
	Yes	6 (1.3)	4 (3.0)	2 (0.6)	
Animation deformity, *n* (%)	No	461 (99.6)	132 (99.2)	329 (99.7)	0.492
	Yes	2 (0.4)	1 (0.8)	1 (0.3)	
Hematoma, *n* (%)	No	455 (98.3)	133 (100.0)	322 (97.6)	0.112
	Yes	8 (1.7)	0 (0.0)	8 (2.4)	
Infection, *n* (%)	No	412 (89.0)	113 (85.0)	299 (90.6)	0.1
	Yes	51 (11.0)	20 (15.0)	31 (9.4)	
Other, *n* (%)	No	457 (98.7)	130 (97.7)	327 (99.1)	0.361
	Yes	6 (1.3)	3 (2.3)	3 (0.9)	
Major complication, *n* (%)	No	441 (95.2)	121 (91.0)	320 (97.0)	0.013
	Yes	22 (4.8)	12 (9.0)	10 (3.0)	
Minor complication, *n* (%)	No	403 (87.0)	110 (82.7)	293 (88.8)	0.092
	Yes	60 (13.0)	23 (17.3)	37 (11.2)	

Major complications include any complications that require surgical intervention; minor complications were defined as any issues that did not necessitate surgical intervention; c-NSM, conventional nipple sparing mastectomy; m-NSM, modified nipple sparing mastectomy; NAC, nipple-areolar complex.

### Aesthetic outcomes using panel assessment scores

Among the 186 breasts with available images obtained in the 6- to 12-month postoperative period evaluated for aesthetic outcomes, the mean panel assessment scores were 2.38 ± 0.95 in c-NSM and 3.14 ± 0.61 in m-NSM (*p *< 0.001) (Table 5). The value of Kendall's W in the inter-reliability analysis was 0.627 for all raters, which shows good reliability. Kendall's W was 0.671 (good reliability) and 0.881 (very good reliability) for general and plastic surgeons, respectively (Table 6).

**Table 5:** Panel assessment by mastectomy type (*N* = 186).

	Overall	c-NSM	m-NSM	*p*-value
	186	48	138	
Overall mean score [mean (SD)]	2.95 (0.79)	2.38 (0.95)	3.14 (0.61)	<0.001
GS 1 [mean (SD)]	3.27 (0.84)	2.75 (0.98)	3.46 (0.71)	<0.001
GS 2 [mean (SD)]	2.56 (1.08)	2.08 (1.20)	2.73 (0.99)	<0.001
GS 3 [mean (SD)]	2.85 (0.96)	2.29 (1.09)	3.04 (0.84)	<0.001
PS 1 [mean (SD)]	3.06 (0.93)	2.40 (1.09)	3.29 (0.75)	<0.001
PS 2 [mean (SD)]	2.99 (1.02)	2.40 (1.16)	3.20 (0.87)	<0.001

c-NSM, conventional nipple sparing mastectomy; m-NSM, modified nipple sparing mastectomy; SD, standard deviation; GS-general surgeon; PS, plastic surgeon.

**Table 6:** Kendall's coefficient of concordance (*N* = 186).

	Kendall's coefficient of concordance W	*p*-value
All surgeons (*n* = 5)	0.627	<0.0001
General surgeons (*n* = 3)	0.671	<0.0001
Plastic surgeons (*n* = 2)	0.881	<0.0001

Additionally, we have performed the cosmetic analysis in terms of reconstruction modalities. The results showed no statistically significant difference among reconstruction methods in terms of panel assessment scores given by general and plastic surgeons in the c-NSM cohort (Supplementary Table S5-1). When we analyzed the c-NSM group in terms of autologous and implant-based reconstructions, one general surgeon (GS 3) has given a higher score to autologous reconstruction, the overall mean scores did not show s significant difference. While the scores given by general surgeons did not show significant differences, plastic surgeons have given the highest scores to reconstruction by TRAM flap and the lowest scores to reconstruction using LD flap in m-NSM cohort (Supplementary Table S5-3). However, it should be noted that the overall mean scores have not differed significantly (*p *= 0.205). The panel assessment scores did not show any statistically significant difference between autologous and implant-based modalities in m-NSM group (Supplementary Table S5-4).

### Patients reported outcomes using breast Q

Only 122 among all patients had complete responses to the questionnaires sent. The time intervals from surgery to Breast Q assessment date were 13.49 and 13.26 months in the c-NSM and m-NSM groups, respectively (*p *= 0.679). The results showed an improved QOL in m-NSM, with higher scores for psychosocial (*p *< 0.001), sexual (*p *= 0.007), and physical well-being (*p *= 0.446). The satisfaction with breast was 68.85 ± 14.12 in c-NSM and 73.21 ± 15.36 in m-NSM (*p *= 0.158) with a minor dominance (Table 7). In addition, the outcomes of Breast Q in terms of the autologous and implant-based reconstruction modalities did not show statistically significant difference in any parts of the questionnaire (Supplementary Tables S7-1, S7-2).

**Table 7:** Breast Q by mastectomy type (*N* = 122).

	Overall	c-NSM	m-NSM	*p*-value
*n*	122	33	89	
Psychosocial well-being [mean (SD)]	79.72 (20.29)	68.21 (23.59)	83.99 (17.20)	<0.001
Sexual well-being [mean (SD)]	61.32 (22.86)	52.79 (24.95)	64.48 (21.33)	0.007
Physical well-being chest [mean (SD)]	27.06 (12.39)	27.76 (10.54)	26.80 (13.06)	0.446
Satisfaction with breast [mean (SD)]	72.03 (15.11)	68.85 (14.12)	73.21 (15.36)	0.158
Satisfaction with information [mean (SD)]	88.20 (15.11)	89.15 (13.79)	87.84 (15.63)	0.651
Satisfaction with surgeon [mean (SD)]	98.56 (6.35)	98.27 (7.80)	98.66 (5.77)	0.765
Interval from surgery, month [mean (SD)]	13.31 (2.84)	13.49 (2.66)	13.26 (2.9)	0.679

c-NSM, conventional nipple sparing mastectomy; m-NSM, modified nipple sparing mastectomy; SD, standard deviation.

### Mastectomy flap thickness

Among patients involved in the study, 41 patients in m-NSM and 37 in c-NSM cohort had a follow up period of longer than 12 months with available postoperative CT scan. The mean ± SD flap thickness was 8.48 ± 1.81 mm in patients who underwent m-NSM, while it was 6.32 ± 1.15 mm in the c-NSM cohort (*p *= 0.02).

## Discussion

Well-established strategies should be applied to prevent ischemic events that necessitate intervention (e.g., debridement, return to the operating room, or even local wound care) and increase the patient burden and healthcare. Obesity, smoking, radiation therapy, the style of mastectomy incision, and the weight of the mastectomy specimen have all been linked to negative outcomes, particularly ischemic problems (16–18). The m-NSM technique showed that the rates of ischemic complications, including partial or total NAC and mastectomy flap necrosis, were lower; however, the values were not statistically significant. This might be due to the retrospective nature of the analysis and collection of complication data from medical records. For instance, partial necrosis, such as small crust formation, may have been missed during charting. Furthermore, the wound healing rate was reduced in the m-NSM group. Our results are consistent with those of previous studies. While it was adopted to maintain the thickness of mastectomy flap at 4–5 mm, increasing the thickness up to 1 cm decreased the ischemic complication rates from over 16% to less than 5%, showing the significant role of thicker flaps in reducing skin necrosis (19–21). Frey et al. analyzed pre- and postoperative breast magnetic resonance imaging (MRIs) and showed that ischemic complications after NSM were significantly associated with thinner postoperative NSM flap thickness (22). In addition, the authors pointed out that the ratio of postoperative to preoperative NSM flap thickness was significantly lower in cases with ischemic complications, and the authors emphasized the importance of dissection at the level of the breast capsule (Cooper ligament plane) during performing NSM. The rate of partial NAC and mastectomy flap necrosis was between 5% and 13%, whereas the rate of full-thickness NAC and mastectomy flap necrosis was between 1% and 7%. Breast cancer is the most frequent cancer in women worldwide and the leading cause of cancer-related deaths in women (23). Mastectomy techniques for breast cancer management have evolved significantly over the years, from radical mastectomy, as described by Halsted, to nipple-sparing mastectomy (NSM). NSM application has expanded dramatically since Freeman's first report in the 1960s (11, 13, 16). NSM allows the plastic surgeon to accomplish a more natural, anatomic, and attractive reconstruction in correctly selected patients by almost completely preserving the breast skin envelope and the NAC (1, 4, 16, 24, 25).

The surgical method used during mastectomy is the most important, although several intrinsic and extrinsic variables influence flap perfusion (26). The most critical aspect may be tissue perfusion of the mastectomy skin flaps, upon which breast reconstruction is conducted (27). Therefore, proper mastectomy flap dissection provides the foundation for successful breast reconstruction. Originally, Camper's fascia was hypothesized to divide subcutaneous fat from the fat surrounding the glandular tissue and to be the “oncoplastic plane” sought by surgeons intending to eve the breast while preserving the skin flap viability (28). However, according to Krohn et al., very thin flaps do not improve oncological safety and are associated with an increased risk of skin necrosis (29). We used modified NSM (m-NSM) to diminish the frequent occurrence of ischemic complications leading to patient dissatisfaction and reduced QOL, in which the anterior lamellar fat layer is preserved to increase the mastectomy flap thickness and perfusion. We dissected on the breast capsule rather than the superficial fascia to achieve a thicker flap, which preserves the anterior lamellar fat with the mastectomy flap by removing only the mammary gland.

We measured the postoperative mastectomy flap thickness using axial CT scan images and found that the mastectomy flap was significantly thicker in breasts operated with m-NSM method than c-NSM (*p *= 0.02). Although the number of patients who have postoperative CT scan with at least 1 year follow-up is limited in both groups, our results showed that preserving the anterior lamellar fat layer by performing m-NSM in patients undergoing IBR increases the flap thickness significantly. Excluding the analysis of patients with less than 12 months follow-up, we avoided the bias related to postoperative swelling.

**Figure 5:**
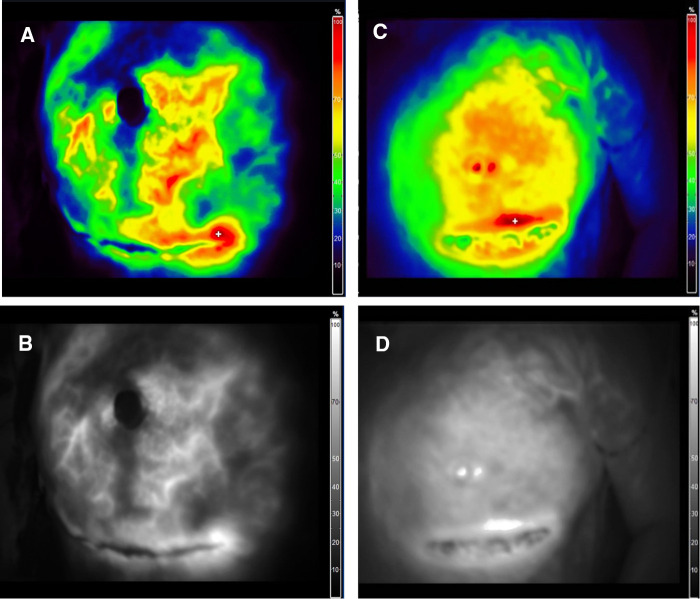
Intraoperative ICG angiography of mastectomy flap. (**A,B**) Color mode and fluorescence angiography of mastectomy flap after c-NSM. (**C,D**) Color mode and fluorescence angiography of mastectomy flap after m-NSM. ICG-indocyanine green.

In addition, our experience with the intraoperative assessment of the mastectomy flap perfusion using ICG angiography shows that the flap perfusion is better retained after m-NSM. Figure 5 illustrates the intraoperative ICG angiography of mastectomy flaps after c-NSM (A,B) and m-NSM (C,D). Both patients were operated through IMF incision. We can see that, the perfusion of the mastectomy flap is notably well-preserved in m-NSM, while the flap after c-NSM is presented with poor perfusion of flap peripheries, upper pole of the flap and the NAC.

These findings revealed that patients who underwent m-NSM followed by IBR presented a significantly lower major complication rate (*p *= 0.013). Moreover, reconstruction failure significantly decreased (*p *= 0.005). While infection and wound healing problems were the main contributing factors to reconstruction failure in the c-NSM group, hematoma was the leading cause of the failure in the m-NSM group. The common hematoma in m-NSM is likely due to better flap perfusion. In most patients requiring immediate hematoma evacuation during the acute postoperative period, the mastectomy flap was the most common source of bleeding.

Advantages of preserving the anterior lamellar fat layer by performing m-NSM are not limited to thicker flap and better perfusion. Because achieving a thicker flap and better perfusion enhances the overall quality of reconstruction. First, this can be explained by the better camouflage of the reconstruction, which makes the reconstructed breast appear closer to a natural breast. The reconstructed breast is very different compared to c-NSM in palpation as one can feel softer and more natural breast. This is due to the preserved fat layer. As we know, the main part of the breast accounts for fatty tissue and thus, the more fat layer is preserved, the more natural the reconstructed breast. Second, as shown in Figure 1, the fat tissue in the upper pole of the breast is preserved in m-NSM while it is excised in c-NSM. That adipose tissue plays a very important role in the reconstruction process. It helps to show the breast naturally by camouflaging the edges of the implant or autologous tissue. The role of the fat grafting after breast augmentation in patients with thin breast skin in improving the cosmetic outcome is well known. The preserved adipose tissue in the upper pole prevents the adjunctive procedures and also if the reconstruction method is autologous flap based, the need for trimming and beveling of the edges is reduced in m-NSM. Third, the postoperative sensation is well-preserved after m-NSM, which increase the sexual satisfaction and quality of life.

More patients in the m-NSM group underwent augmentation mammoplasty of the opposite breast (*p *< 0.001). During preoperative consultation, many patients in this group asked for simultaneous augmentation mammoplasty of the opposite breast at the time of reconstruction. Conversely, it is difficult to achieve a volumetric balance of the two breasts after m-NSM in Korean patients when reconstruction is performed using implants because of the smaller weight of the excised glandular tissue, making it impossible to find the implant in that size.

The m-NSM was associated with superior cosmetic outcomes according to the data obtained using panel assessment scores. The overall average score for m-NSM was 3.14, and 2.38 in the c-NSM group (*p *< 0.001). The scores by the general and plastic surgeons were almost the same in both groups. Moreover, the analysis of cosmetic outcomes by reconstruction modalities did not show a significant difference between the overall mean scores (Supplementary Tables S5-1,2,3,4).

A more reliable estimate can be made when patient-reported outcomes are combined with aesthetic evaluations performed by health professionals, as the primary justification for breast reconstruction has a positive impact on patient QOL following mastectomy (30). Furthermore, although evaluating breast aesthetics by panel assessment alone is possible, using patient-reported outcomes based on Breast-Q allows evaluation of the functional and psychological aspects of breast reconstruction, which are unknown to medical professionals. Compared with c-NSM, patients in the m-NSM group reported significantly higher psychosocial (*p *< 0.001) and sexual (*p *= 0.007) well-being. Psychosocial dominance may be explained by higher self-confidence in m-NSM due to the more natural appearance of the breasts. Better sexual well-being might result from better sensation on the breast skin, particularly NAC, which is less compromised in m-NSM. However, the satisfaction domain of the Breast Q did not show a significant difference between the groups.

We have included the patient only if they have completed the Breast Q questionnaire. Also, we have included the patients for the aesthetic outcome analysis if they have a postoperative image. Unfortunately, the quite considerable number of patients did not answer the Breast Q questionnaire and did not have a postoperative image to perform the aesthetic analysis. However, considering that the overall number of included patients was big enough, including all of them in the study, which involves multiple investigations (complication, aesthetic, patients reported and oncological) is difficult. In addition, all eligible patients were involved in the aesthetic as well as PRO studies randomly, without intentional grouping. Therefore, we can say that our results have a significant role in improving the outcomes of breast reconstruction.

The m-NSM was associated with statistically similar recurrence rates with c-NSM, although the follow-up periods were too short to conclude about oncological safety. The rates of local (*p *= 0.209) and distal (*p *= 0.504) recurrences were lower in m-NSM, although the difference was not statistically significant. At first glance, this might be due to the follow-up period of m-NSM being shorter than that of c-NSM, 31.98 and 41.92 months, respectively; *p *< 0.001). Second, the additional superficial margin was the plane of dissection in patients with a tumor located close to the breast capsule.

This study had a few limitations. First, it was a retrospective study. Moreover, as c-NSM application in our hospital started earlier, the mean follow-up period was shorter in the m-NSM group. This may be associated with a better mastectomy flap quality in more recent cases due to the improvement in operating surgeons` experience. Second, we included only patients who did not receive PMRT because the number of patients in the c-NSM group was insufficient for statistical analysis. Third, the number of patients with available postoperative CT scan as well as enough follow-up was limited and the application of CT for the objective assessment is less accurate compared to MRI. Finally, oncological safety is considered a very important part of the breast cancer treatment. However, the follow-up period for making any conclusion about the oncological safety of the method is not enough (41.92 and 31.98 months for c-NSM and m-NSM groups, respectively). This is one of the major limitations of our study. Although the follow-up period is short, we have performed an oncological safety assessment as additional data. Because the follow-up period is insufficient to prove the technique's oncologic safety, further studies involving a longer follow-up period are needed. Despite the limitations mentioned above, the findings of this study can guide surgeons in maximizing the outcomes of breast reconstruction while reducing the associated complications and improving the QOL, considering that providing the best possible reconstruction to improve QOL and patient satisfaction is vitally valuable, although the primary purpose of the procedure is to treat cancer.

Although more research is needed to assess oncologic safety, preserving the anterior lamellar fat during m-NSM for IBR in our study was associated with overall lower rates of complications, including ischemia of the mastectomy flap as well as NAC, better aesthetic outcomes, and QOL.

## Data Availability

The original contributions presented in the study are included in the article/**Supplementary Material**, further inquiries can be directed to the corresponding author/s.
