# Supplementary material for: Conventional versus modified nipple sparing mastectomy in immediate breast reconstruction: Complications, aesthetic, and patient-reported outcomes
Source: Front Surg. 2022 Sep 21;9:1001019. doi: 10.3389/fsurg.2022.1001019 (PMC9583667; doi:10.3389/fsurg.2022.1001019)
Supplement: Supplementary file 1 [file DataSheet1.docx]

**Supplementary material**

| **Table 4-1: Complication by Reconstruction type (c-NSM only, N=133)** | | | | | | | |
| --- | --- | --- | --- | --- | --- | --- | --- |
|  | **Level** | **Overall** | **TRAM** | **DTI** | **TEI** | **LD** | ***p*-value** |
| **n** |  | 133 | 56 | 26 | 48 | 3 |  |
| Partial skin necrosis, n(%) | No | 131 (98.5) | 55 (98.2) | 26 (100.0) | 47 (97.9) | 3 (100.0) | 1 |
|  | Yes | 2 (1.5) | 1 (1.8) | 0 (0.0) | 1 (2.1) | 0 (0.0) |  |
| Partial NAC necrosis, n(%) | No | 130 (97.7) | 55 (98.2) | 26 (100.0) | 46 (95.8) | 3 (100.0) | 0.625 |
|  | Yes | 3 (2.3) | 1 (1.8) | 0 (0.0) | 2 (4.2) | 0 (0.0) |  |
| Total NAC necrosis, n(%) | No | 132 (99.2) | 55 (98.2) | 26 (100.0) | 48 (100.0) | 3 (100.0) | 1 |
|  | Yes | 1 (0.8) | 1 (1.8) | 0 (0.0) | 0 (0.0) | 0 (0.0) |  |
| Wound healing problem, n(%) | No | 128 (96.2) | 54 (96.4) | 25 (96.2) | 46 (95.8) | 3 (100.0) | 1 |
|  | Yes | 5 (3.8) | 2 (3.6) | 1 (3.8) | 2 (4.2) | 0 (0.0) |  |
| Seroma, n(%) | No | 131 (98.5) | 56 (100.0) | 24 (92.3) | 48 (100.0) | 3 (100.0) | 0.082 |
|  | Yes | 2 (1.5) | 0 (0.0) | 2 (7.7) | 0 (0.0) | 0 (0.0) |  |
| Reconstruction failure, n(%) | No | 124 (93.2) | 53 (94.6) | 25 (96.2) | 43 (89.6) | 3 (100.0) | 0.631 |
|  | Yes | 4 (3.0) | 0 (0.0) | 4 (15.4) | 0 (0.0) | 0 (0.0) |  |
| Animation deformity, n(%) | No | 132 (99.2) | 56 (100.0) | 25 (96.2) | 48 (100.0) | 3 (100.0) | 0.218 |
|  | Yes | 1 (0.8) | 0 (0.0) | 1 (3.8) | 0 (0.0) | 0 (0.0) |  |
| Hematoma, n(%) | No | 133 (100.0) | 56 (100.0) | 26 (100.0) | 48 (100.0) | 3 (100.0) | NA |
| Infection, n(%) | No | 113 (85.0) | 51 (91.1) | 21 (80.8) | 38 (79.2) | 3 (100.0) | 0.287 |
|  | Yes | 20 (15.0) | 5 (8.9) | 5 (19.2) | 10 (20.8) | 0 (0.0) |  |
| Other, n(%) | No | 130 (97.7) | 55 (98.2) | 25 (96.2) | 47 (97.9) | 3 (100.0) | 0.807 |
|  | Yes | 3 (2.3) | 1 (1.8) | 1 (3.8) | 1 (2.1) | 0 (0.0) |  |
| Major complication, n(%) | No | 121 (91.0) | 52 (92.9) | 23 (88.5) | 43 (89.6) | 3 (100.0) | 0.841 |
|  | Yes | 12 (9.0) | 4 (7.1) | 3 (11.5) | 5 (10.4) | 0 (0.0) |  |
| Minor complication, n(%) | No | 110 (82.7) | 50 (89.3) | 18 (69.2) | 39 (81.2) | 3 (100.0) | 0.155 |
|  | Yes | 23 (17.3) | 6 (10.7) | 8 (30.8) | 9 (18.8) | 0 (0.0) |  |

| **Table 4-2: Complication by Reconstruction type (m-NSM only, N=330)** | | | | | | | |
| --- | --- | --- | --- | --- | --- | --- | --- |
|  | **Level** | **Overall** | **TRAM** | **DTI** | **TEI** | **LD** | ***p*-value** |
| **n** |  | 330 | 125 | 82 | 82 | 41 |  |
| Partial skin necrosis, n(%) | No | 330 (100.0) | 125 (100.0) | 82 (100.0) | 82 (100.0) | 41 (100.0) | NA |
| Partial NAC necrosis, n(%) | No | 329 (99.7) | 124 (99.2) | 82 (100.0) | 82 (100.0) | 41 (100.0) | 1 |
|  | Yes | 1 (0.3) | 1 (0.8) | 0 (0.0) | 0 (0.0) | 0 (0.0) |  |
| Total NAC necrosis, n(%) | No | 330 (100.0) | 125 (100.0) | 82 (100.0) | 82 (100.0) | 41 (100.0) | NA |
| Wound healing problem, n(%) | No | 328 (99.4) | 124 (99.2) | 82 (100.0) | 81 (98.8) | 41 (100.0) | 1 |
|  | Yes | 2 (0.6) | 1 (0.8) | 0 (0.0) | 1 (1.2) | 0 (0.0) |  |
| Seroma, n(%) | No | 327 (99.1) | 125 (100.0) | 81 (98.8) | 82 (100.0) | 39 (95.1) | 0.024 |
|  | Yes | 3 (0.9) | 0 (0.0) | 1 (1.2) | 0 (0.0) | 2 (4.9) |  |
| Reconstruction failure, n(%) | No | 325 (98.5) | 124 (99.2) | 82 (100.0) | 78 (95.1) | 41 (100.0) | 0.049 |
|  | Yes | 2 (0.6) | 0 (0.0) | 2 (2.4) | 0 (0.0) | 0 (0.0) |  |
| Animation deformity, n(%) | No | 329 (99.7) | 125 (100.0) | 82 (100.0) | 82 (100.0) | 40 (97.6) | 0.124 |
|  | Yes | 1 (0.3) | 0 (0.0) | 0 (0.0) | 0 (0.0) | 1 (2.4) |  |
| Hematoma, n(%) | No | 322 (97.6) | 122 (97.6) | 82 (100.0) | 78 (95.1) | 40 (97.6) | 0.226 |
|  | Yes | 8 (2.4) | 3 (2.4) | 0 (0.0) | 4 (4.9) | 1 (2.4) |  |
| Infection, n(%) | No | 299 (90.6) | 112 (89.6) | 79 (96.3) | 69 (84.1) | 39 (95.1) | 0.042 |
|  | Yes | 31 (9.4) | 13 (10.4) | 3 (3.7) | 13 (15.9) | 2 (4.9) |  |
| Other, n(%) | No | 327 (99.1) | 124 (99.2) | 81 (98.8) | 81 (98.8) | 41 (100.0) | 1 |
|  | Yes | 3 (0.9) | 1 (0.8) | 1 (1.2) | 1 (1.2) | 0 (0.0) |  |
| Major complication, n(%) | No | 320 (97.0) | 120 (96.0) | 82 (100.0) | 77 (93.9) | 41 (100.0) | 0.064 |
|  | Yes | 10 (3.0) | 5 (4.0) | 0 (0.0) | 5 (6.1) | 0 (0.0) |  |
| Minor complication, n(%) | No | 293 (88.8) | 111 (88.8) | 75 (91.5) | 69 (84.1) | 38 (92.7) | 0.435 |
|  | Yes | 37 (11.2) | 14 (11.2) | 7 (8.5) | 13 (15.9) | 3 (7.3) |  |

| **Table 4-3: Complication by Autologous vs. Implant (c-NSM only, N=133)** | | | | | |
| --- | --- | --- | --- | --- | --- |
|  | **level** | **Overall** | **Autologous** | **Implant** | ***p*-value** |
| **n** |  | 133 | 59 | 74 |  |
| Partial skin necrosis, n(%) | No | 131 (98.5) | 58 (98.3) | 73 (98.6) | 1 |
|  | Yes | 2 (1.5) | 1 (1.7) | 1 (1.4) |  |
| Partial NAC necrosis, n(%) | No | 130 (97.7) | 58 (98.3) | 72 (97.3) | 1 |
|  | Yes | 3 (2.3) | 1 (1.7) | 2 (2.7) |  |
| Total NAC necrosis, n(%) | No | 132 (99.2) | 58 (98.3) | 74 (100.0) | 0.444 |
|  | Yes | 1 (0.8) | 1 (1.7) | 0 (0.0) |  |
| Wound healing problem, n(%) | No | 128 (96.2) | 57 (96.6) | 71 (95.9) | 1 |
|  | Yes | 5 (3.8) | 2 (3.4) | 3 (4.1) |  |
| Seroma, n(%) | No | 131 (98.5) | 59 (100.0) | 72 (97.3) | 0.503 |
|  | Yes | 2 (1.5) | 0 (0.0) | 2 (2.7) |  |
| Reconstruction failure, n(%) | No | 124 (93.2) | 56 (94.9) | 68 (91.9) | 0.731 |
|  | Yes | 4 (3.0) | 0 (0.0) | 4 (5.4) |  |
| Animation deformity, n(%) | No | 132 (99.2) | 59 (100.0) | 73 (98.6) | 1 |
|  | Yes | 1 (0.8) | 0 (0.0) | 1 (1.4) |  |
| Hematoma, n(%) | No | 133 (100.0) | 59 (100.0) | 74 (100.0) | NA |
| Infection, n(%) | No | 113 (85.0) | 54 (91.5) | 59 (79.7) | 0.086 |
|  | Yes | 20 (15.0) | 5 (8.5) | 15 (20.3) |  |
| Other, n(%) | No | 130 (97.7) | 58 (98.3) | 72 (97.3) | 1 |
|  | Yes | 3 (2.3) | 1 (1.7) | 2 (2.7) |  |
| Major complication, n(%) | No | 121 (91.0) | 55 (93.2) | 66 (89.2) | 0.548 |
|  | Yes | 12 (9.0) | 4 (6.8) | 8 (10.8) |  |
| Minor complication, n(%) | No | 110 (82.7) | 53 (89.8) | 57 (77.0) | 0.066 |
|  | Yes | 23 (17.3) | 6 (10.2) | 17 (23.0) |  |

| **Table 4-4: Complication by Autologous vs. Implant (m-NSM only, N=330)** | | | | | |
| --- | --- | --- | --- | --- | --- |
|  | **level** | **Overall** | **Autologous** | **Implant** | ***p*-value** |
| **n** |  | 330 | 166 | 164 |  |
| Partial skin necrosis, n(%) | No | 330 (100.0) | 166 (100.0) | 164 (100.0) | NA |
| Partial NAC necrosis, n(%) | No | 329 (99.7) | 165 (99.4) | 164 (100.0) | 1 |
|  | Yes | 1 (0.3) | 1 (0.6) | 0 (0.0) |  |
| Total NAC necrosis, n(%) | No | 330 (100.0) | 166 (100.0) | 164 (100.0) | NA |
| Wound healing problem, n(%) | No | 328 (99.4) | 165 (99.4) | 163 (99.4) | 1 |
|  | Yes | 2 (0.6) | 1 (0.6) | 1 (0.6) |  |
| Seroma, n(%) | No | 327 (99.1) | 164 (98.8) | 163 (99.4) | 1 |
|  | Yes | 3 (0.9) | 2 (1.2) | 1 (0.6) |  |
| Reconstruction failure, n(%) | No | 325 (98.5) | 165 (99.4) | 160 (97.6) | 0.213 |
|  | Yes | 2 (0.6) | 0 (0.0) | 2 (1.2) |  |
| Animation deformity, n(%) | No | 329 (99.7) | 165 (99.4) | 164 (100.0) | 1 |
|  | Yes | 1 (0.3) | 1 (0.6) | 0 (0.0) |  |
| Hematoma, n(%) | No | 322 (97.6) | 162 (97.6) | 160 (97.6) | 1 |
|  | Yes | 8 (2.4) | 4 (2.4) | 4 (2.4) |  |
| Infection, n(%) | No | 299 (90.6) | 151 (91.0) | 148 (90.2) | 0.852 |
|  | Yes | 31 (9.4) | 15 (9.0) | 16 (9.8) |  |
| Other, n(%) | No | 327 (99.1) | 165 (99.4) | 162 (98.8) | 0.622 |
|  | Yes | 3 (0.9) | 1 (0.6) | 2 (1.2) |  |
| Major complication, n(%) | No | 320 (97.0) | 161 (97.0) | 159 (97.0) | 1 |
|  | Yes | 10 (3.0) | 5 (3.0) | 5 (3.0) |  |
| Minor complication, n(%) | No | 293 (88.8) | 149 (89.8) | 144 (87.8) | 0.604 |
|  | Yes | 37 (11.2) | 17 (10.2) | 20 (12.2) |  |

| **Table 5-1: Panel assessment by Reconstruction type (c-NSM only, N= 48)** | | | | | | | |
| --- | --- | --- | --- | --- | --- | --- | --- |
|  |  | **Overall** | **TRAM** | **DTI** | **TEI** | **LD** | ***p*-value** |
|  |  | 48 | 10 | 7 | 29 | 2 |  |
| Overall mean score  (mean (SD)) |  | 2.38 (0.95) | 2.64 (1.32) | 2.29 (1.13) | 2.31 (0.79) | 2.50 (0.71) | 0.68 |
| GS 1 (mean (SD)) |  | 2.75 (0.98) | 2.70 (1.42) | 2.86 (1.21) | 2.69 (0.76) | 3.50 (0.71) | 0.621 |
| GS 2 (mean (SD)) |  | 2.08 (1.20) | 2.20 (1.40) | 1.86 (1.07) | 2.07 (1.22) | 2.50 (0.71) | 0.799 |
| GS 3 (mean (SD)) |  | 2.29 (1.09) | 2.80 (1.40) | 2.43 (1.27) | 2.03 (0.87) | 3.00 (1.41) | 0.191 |
| PS 1 (mean (SD)) |  | 2.40 (1.09) | 2.80 (1.48) | 2.00 (1.15) | 2.38 (0.94) | 2.00 (0.00) | 0.414 |
| PS 2 (mean (SD)) |  | 2.40 (1.16) | 2.70 (1.34) | 2.29 (1.38) | 2.38 (1.08) | 1.50 (0.71) | 0.529 |
| c-NSM=conventional Nipple sparing mastectomy; SD=standard deviation; GS-general surgeon; PS=plastic surgeon. | | | | | | | |

| **Table 5-2: Panel assessment by Autologous vs. Implant (c-NSM only, N= 48)** | | | | | |
| --- | --- | --- | --- | --- | --- |
|  |  | **Overall** | **Autologous** | **Implant** | ***p*-value** |
|  |  | 48 | 12 | 36 |  |
| Overall mean score  (mean (SD)) |  | 2.38 (0.95) | 2.62 (1.21) | 2.31 (0.85) | 0.232 |
| GS 1 (mean (SD)) |  | 2.75 (0.98) | 2.83 (1.34) | 2.72 (0.85) | 0.446 |
| GS 2 (mean (SD)) |  | 2.08 (1.20) | 2.25 (1.29) | 2.03 (1.18) | 0.436 |
| GS 3 (mean (SD)) |  | 2.29 (1.09) | 2.83 (1.34) | 2.11 (0.95) | 0.042 |
| PS 1 (mean (SD)) |  | 2.40 (1.09) | 2.67 (1.37) | 2.31 (0.98) | 0.297 |
| PS 2 (mean (SD)) |  | 2.40 (1.16) | 2.50 (1.31) | 2.36 (1.13) | 0.649 |
| c-NSM=modified Nipple sparing mastectomy; SD=standard deviation; GS-general surgeon; PS=plastic surgeon. | | | | | |

| **Table 5-3: Panel assessment by Reconstruction type (m-NSM only, N=138)** | | | | | | | |
| --- | --- | --- | --- | --- | --- | --- | --- |
|  |  | **Overall** | **TRAM** | **DTI** | **TEI** | **LD** | ***p*-value** |
|  |  | 138 | 47 | 32 | 32 | 27 |  |
| Overall mean score  (mean (SD)) |  | 3.14 (0.61) | 3.24 (0.57) | 3.12 (0.60) | 3.21 (0.60) | 2.93 (0.68) | 0.205 |
| GS 1 (mean (SD)) |  | 3.46 (0.71) | 3.45 (0.65) | 3.25 (0.84) | 3.72 (0.46) | 3.41 (0.80) | 0.113 |
| GS 2 (mean (SD)) |  | 2.73 (0.99) | 2.60 (0.90) | 2.81 (1.09) | 2.84 (1.02) | 2.74 (1.02) | 0.635 |
| GS 3 (mean (SD)) |  | 3.04 (0.84) | 3.28 (0.71) | 3.00 (0.76) | 2.94 (0.88) | 2.81 (1.00) | 0.148 |
| PS 1 (mean (SD)) |  | 3.29 (0.75) | 3.45 (0.75) | 3.38 (0.66) | 3.34 (0.79) | 2.85 (0.66) | 0.004 |
| PS 2 (mean (SD)) |  | 3.20 (0.87) | 3.45 (0.77) | 3.19 (0.78) | 3.19 (0.97) | 2.81 (0.92) | 0.018 |
| m-NSM=modified Nipple sparing mastectomy; SD=standard deviation; GS-general surgeon; PS=plastic surgeon. | | | | | | | |

| **Table 5-4: Panel assessment by Autologous vs. Implant (m-NSM only, N=138)** | | | | | |
| --- | --- | --- | --- | --- | --- |
|  |  | **Overall** | **Autologous** | **Implant** | **P-value** |
|  |  | 138 | 74 | 64 |  |
| Overall mean score  (mean (SD)) |  | 3.14 (0.61) | 3.13 (0.63) | 3.17 (0.60) | 0.625 |
| GS 1 (mean (SD)) |  | 3.46 (0.71) | 3.43 (0.70) | 3.48 (0.71) | 0.603 |
| GS 2 (mean (SD)) |  | 2.73 (0.99) | 2.65 (0.94) | 2.83 (1.05) | 0.257 |
| GS 3 (mean (SD)) |  | 3.04 (0.84) | 3.11 (0.85) | 2.97 (0.82) | 0.254 |
| PS 1 (mean (SD)) |  | 3.29 (0.75) | 3.23 (0.77) | 3.36 (0.72) | 0.327 |
| PS 2 (mean (SD)) |  | 3.20 (0.87) | 3.22 (0.88) | 3.19 (0.87) | 0.796 |
| m-NSM=modified Nipple sparing mastectomy; SD=standard deviation; GS-general surgeon; PS=plastic surgeon. | | | | | |

| **Table 7-1: Breast Q by Auto vs. Implant (c-NSM only; N=33)** | | | | |
| --- | --- | --- | --- | --- |
|  | **Overall** | **Autologous** | **Implant** | ***p*-value** |
| n | 33 | 19 | 14 |  |
| Psychosocial well-being (mean (SD)) | 68.21 (23.59) | 69.32 (25.09) | 66.71 (22.22) | 0.609 |
| Sexual well-being (mean (SD)) | 52.79 (24.95) | 49.74 (22.87) | 56.93 (27.85) | 0.882 |
| Physical well-being chest (mean (SD)) | 27.76 (10.54) | 28.84 (12.85) | 26.29 (6.37) | 0.74 |
| Satisfaction with breast (mean (SD)) | 68.85 (14.12) | 67.95 (13.62) | 70.07 (15.20) | 0.756 |
| Satisfaction with information (mean (SD)) | 89.15 (13.79) | 87.16 (15.56) | 91.86 (10.92) | 0.444 |
| Satisfaction with surgeon (mean (SD)) | 98.27 (7.80) | 97.00 (10.20) | 100.00 (0.00) | 0.218 |

| **Table 7-2: Breast Q by Auto vs. Implant (m-NSM only; N=89)** | | | | |
| --- | --- | --- | --- | --- |
|  | **Overall** | **Autologous** | **Implant** | ***p*-value** |
| n | 89 | 51 | 38 |  |
| Psychosocial well-being (mean (SD)) | 83.99 (17.20) | 82.55 (18.44) | 85.92 (15.40) | 0.528 |
| Sexual well-being (mean (SD)) | 64.48 (21.33) | 62.88 (21.80) | 66.63 (20.77) | 0.263 |
| Physical well-being chest (mean (SD)) | 26.80 (13.06) | 29.02 (12.87) | 23.82 (12.88) | 0.071 |
| Satisfaction with breast (mean (SD)) | 73.21 (15.36) | 71.00 (14.68) | 76.18 (15.94) | 0.092 |
| Satisfaction with information (mean (SD)) | 87.84 (15.63) | 85.43 (14.16) | 91.08 (17.07) | 0.095 |
| Satisfaction with surgeon (mean (SD)) | 98.66 (5.77) | 98.25 (7.23) | 99.21 (2.85) | 0.957 |
